# Computational workflow to study the seasonal variation of secondary metabolites in nine different bryophytes

**DOI:** 10.1038/sdata.2018.179

**Published:** 2018-08-28

**Authors:** Kristian Peters, Karin Gorzolka, Helge Bruelheide, Steffen Neumann

**Affiliations:** 1 Leibniz Institute of Plant Biochemistry, Stress and Developmental Biology, Weinberg 3, 06120 Halle (Saale), Germany; 2 Institute of Biology/Geobotany and Botanical Garden, Martin Luther University Halle Wittenberg, Am Kirchtor 1, 06108 Halle (Saale), Germany; 3 German Centre for Integrative Biodiversity Research (iDiv) Halle-Jena-Leipzig, Deutscher Platz 5e, 04103 Leipzig, Germany

**Keywords:** Metabolomics, Ecophysiology, Scientific data, Plant sciences

## Abstract

In Eco-Metabolomics interactions are studied of non-model organisms in their natural environment and relations are made between biochemistry and ecological function. Current challenges when processing such metabolomics data involve complex experiment designs which are often carried out in large field campaigns involving multiple study factors, peak detection parameter settings, the high variation of metabolite profiles and the analysis of non-model species with scarcely characterised metabolomes. Here, we present a dataset generated from 108 samples of nine bryophyte species obtained in four seasons using an untargeted liquid chromatography coupled with mass spectrometry acquisition method (LC/MS). Using this dataset we address the current challenges when processing Eco-Metabolomics data. Here, we also present a reproducible and reusable computational workflow implemented in Galaxy focusing on standard formats, data import, technical validation, feature detection, diversity analysis and multivariate statistics. We expect that the representative dataset and the reusable processing pipeline will facilitate future studies in the research field of Eco-Metabolomics.

## Background & Summary

In Ecological Metabolomics (or short “Eco-Metabolomics”), metabolite profiles of organisms are studied in order to describe ecological processes such as biotic interactions or the impact of environmental changes on various biological species^1–3^. In contrast to biochemistry, wild non-model species are typically studied in their natural environment in ecology. This often involves different individuals of one or more species from populations growing under quite heterogeneous conditions when compared to the controlled conditions in greenhouses or growth chambers. As a result, metabolite profiles are highly variable when compared to each other. Moreover, profiles of non-model species contain a large number of novel compounds (so called “unknown unknowns”) that are difficult to identify because of lacking reference compounds, which have so far been mostly elucidated in model organisms^3,4^. Furthermore, designing ecological experiments is often complex and involves multiple factors^5^. Thus, the metabolomics data processing pipeline needs to be adapted in order to deal with the particular hypotheses and idiosyncrasies of ecological experiments.

Here, we present a descriptor for a dataset that we consider representative for the research field of Eco-Metabolomics. Our study makes use of a field campaign with a two-factorial design (seasons and species), which includes (except *Marchantia polymorpha*) non-model species of bryophytes. In order to facilitate subsequent analysis, we kept the experiment design as simple as possible. The sampling was conducted on-site at the Botanical Garden of Martin Luther University Halle-Wittenberg once in each season over a period of one year (see below). Metabolite profiles were acquired using untargeted liquid chromatography coupled with mass spectrometry (LC/MS). Raw metabolite profiles are available in the metabolomics data repository MetaboLights^6^ (Data Citation 1).

**Figure 1:**
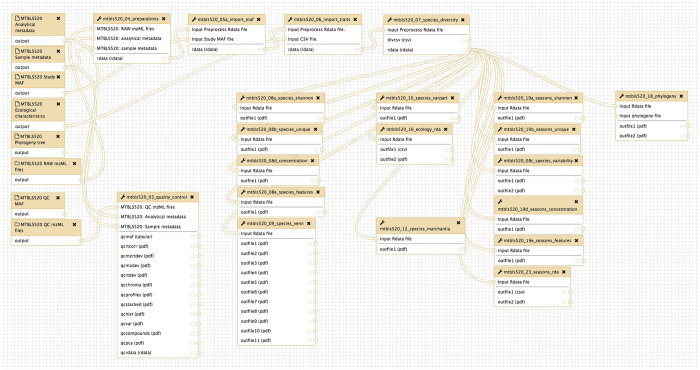
Computational workflow of the whole study (Data Citation 1) running in the Galaxy Workflow Management system. Each of the modules represent a particular step in the study of Peters *et al.*^9^. The modules have defined inputs, outputs and sets of parameters. The modules are connected to each other to give the resulting workflow. The function of the modules is explained in Table 1 (available online only).

In biochemistry there are strict laboratory protocols that ensure reproducibility of the analytical methods, while in bioinformatics this function is accomplished by implementing reusable computational workflows^7,8^. Thus, in addition to the dataset we also address the typical bioinformatic challenges that come with Eco-Metabolomics experiments by implementing a reproducible and reusable computational workflow (Fig. 1). While the analysis and ecological interpretation of the study is described in Peters *et al.*^9^, here we focus on the analytical and bioinformatic work that is required to create a computational processing pipeline that is reproducible and that can be reused by other subsequent studies.

We describe in detail the experimental methodology that was used to create the dataset as well as the methodology to make the computational workflow reproducible (to give identical results in different computational environments). By formalizing and validating the processes that led to the results^10,11^, we expect that this approach can serve as a model for subsequent studies. We further expect that Eco-Metabolomics studies use our dataset and the computational workflow to foster reuse and improve future data processing pipelines.

## Methods

These methods describe in detail the steps in producing the data, including full descriptions of the experimental design in our related work^9^, data acquisition, computational processing, diversity analysis, biostatistics and bioinformatics procedures.

### Sampling campaign

Samples of the nine moss species Brachythecium rutabulum (Hedw.) Schimp., Calliergonella cuspidata (Hedw.) Loeske, Fissidens taxifolius Hedw., Grimmia pulvinata (Hedw.) Sm., Hypnum cupressiforme Hedw. (H. lacunosum was not differentiated), Marchantia polymorpha L., Plagiomnium undulatum (Hedw.) T.J. Kop., Polytrichum strictum Menzies ex Brid. and Rhytidiadelphus squarrosus (Hedw.) Warnst. were collected in the Botanical Gardens of the Martin-Luther-University Halle-Wittenberg, Germany. Sampling was performed in summer (2016/08/08), autumn (2016/11/09), winter (2017/01/27) and spring (2017/05/11) at relatively stable weather conditions as it is known that short-term climatic fluctuations and rainfall can influence secondary metabolite content and ammonium uptake of bryophytes^12^. Thus, the bryophytes were only collected when there was sunshine at least two days prior to and during sampling. Furthermore, sampling was performed after mid-day between 13:00 and 15:00.

### Sampling protocol

In each season, three composite samples of different individuals of each species were taken, leading to a total of 3 * 9 * 4=108 samples. Only above-ground parts of the moss gametophytes such as leaves, branches, stems or thalloid parts were taken for sampling. From dioecious species such as *M. polymorpha*, *P. strictum* and *P. undulatum* female, male and sterile gametophytes were collected in a composite sample. Before sampling, visible archegonial and antheridial heads and any belowground parts such as rhizoids and rooting stems were removed with a sterile tweezer. The gametophytic moss parts were put in Eppendorf tubes and were frozen instantly on dry ice and later in the lab in liquid nitrogen.

### Collecting ecological characteristics

In order to relate metabolomes of the bryophytes to ecology, several ecological characteristics were recorded on-site and compiled from literature. The on-site characteristics *type of substrate* with the nominal/categorical levels “soil”, “rock with lean soil cover” and “rock”; *light conditions* with the ordinal levels “sunny”, “half-shade” and “shade”; *moisture of the substrate* with the ordinal levels “dry”, “fresh”, “damp” and wet; and *exposition* with the nominal levels “North”, “East”, “South”, “West”, “Northeast”, “Northwest”, “Southeast” and “Southwest” were recorded when taking the samples in the field.

The nominal characteristics *growth form*, *habitat type, substrate* and *life strategy*, the ordinal life-history characteristics *spore size*, *gametangia distribution* and *sexual reproduction frequency*, as well as the ordinal Ellenberg indicator values (indices for *light*, *temperature*, *continentality*, *moisture*, *reaction*, *nitrogen* and *life-form*) were collected from the literature^13–17^. For an overview, please refer Table 1 (available online only) in Peters *et al.*^9^ or the file m_characteristics.csv in the dataset (see Data Citation 1, and Table 1 (available online only)).

### Extraction protocol and LC/MS analysis

Frozen moss samples were homogenized by adding 200 mg ceramic beads (0.5 mm diameter, Roth) and ribolysing (Precellys 24, 2×20 s at 6500 r.p.m., 5 min pause in liquid nitrogen). 1 ml ice-cold 80/20 (v/v) methanol/water spiked with internal standards 5 μM biochanin A (Sigma-Aldrich), 5 μM kinetin (Sigma-Aldrich) and 5 μM N-(3-indolylacetyl)-l-valine (Sigma-Aldrich) were added. Samples were vortexed and thawed while shaking for 15 min at 1,000 r.p.m. at room temperature followed by ultrasonification for 15 min and again 15 min shaking. After 15 min centrifugation at 13,000 r.p.m. 500 μl of supernatant were dried in a vacuum centrifuge at 40 °C and reconstituted in 80/20 (v/v) methanol/water with the volume adjusted to the initial fresh weight of the sample to a final concentration of 10 mg fresh weight per 100 μl extract.

Chromatographic separations were performed at 40 °C on an Acquity UPLC system (Waters) equipped with an HSS T3 column (100×1 mm, particle size 1.8 μm; Waters) applying the following binary gradient at a flow rate of 150 μL min^−1^: 0 to 1 min, isocratic 95% A (water:formic acid: 99.9:0.1 [v/v]), 5% B (acetonitrile:formic acid: 99.9:0.1 [v/v]); 1 to 18 min, linear from 5 to 95% B; 18 to 20 min, isocratic 95% B. The injection volume was 2.0 μL (full loop injection).

Ultra-performance liquid chromatography coupled to electrospray ionization quadrupole time-of-flight mass spectrometry (UPLC/ESI-QTOF-MS) was performed using a high resolution MicrOTOF-Q II hybrid quadrupole time-of-flight mass spectrometer^18^. Data were acquired with the following MS instrument settings: nebulizer gas: nitrogen, 1.4 bar; dry gas: nitrogen, 6 L min^−1^, 190 °C; capillary: 5000 V (+4000 V for negative mode); end plate offset: −500 V; funnel 1 radio frequency (RF): 200 Volts peak-to-peak (Vpp); funnel 2 RF: 200 Vpp; in-source collision-induced dissociation (CID) energy: 10 eV; hexapole RF: 100 Vpp; quadrupole ion energy: 3 eV (−5 eV for neg-mode); collision gas: nitrogen; collision energy: 7 eV (−7 eV for negative mode); collision cell RF: 250 Vpp (150 Vpp for negative mode); transfer time: 70 μs; prepulse storage: 5 μs; pulser frequency: 10 kHz; and spectra rate: 3 Hz. Mass spectra were acquired in centroid mode. Calibration of the m/z scale was performed for individual raw data files on lithium formate cluster ions obtained by automatic infusion of 20 μL of 10 mM lithium hydroxide in isopropanol:water:formic acid, 49.9:49.9:0.2 (v/v/v) at the end of the gradient.

### Quality control

In order to validate the instrument performance and to detect batch effects between the instrument runs, the following quality control (QC) protocol was realized. Samples with a lab-internal standard mix (MM8) were interspersed before and after 7 bryophyte samples in the MicrOTOF^18^. The following substances were used in the MM8: 2-Phenylglycine (Fluka), Kinetin (Roth), Rutin (Acros Organics), O-Methylsalicylic acid (Sigma), Phlorizin dihydrate (Sigma), N-(3-Indolyacetyl)-L-valine (Sigma), 3-Indolylacetonitrile (Fluka) and Biochanin A (Sigma). Substances in the MM8 were selected based on their ionization properties (ionization in both positive and negative mode and the differential adduct formation) and a wide coverage of known retention times throughout the gradient with our instrumental setup. Known ionization properties were used to detect shifts and effects in mass-to-charge ratios (m/z) and retention times (RT) of the respective batches and to validate RT correction made by XCMS (see below).

### Raw data acquisition

Raw LC/MS data were converted to the open data format mzML^19^ with the software CompassXPort 3.0.9 from Bruker Daltonics (available at http://www.bruker.com/service/support-upgrades/software-downloads.html). In compliance with the minimum information guidelines for Metabolomics studies^20^, metadata were recorded to ISA-Tab format^21^ using ISAcreator 1.7.10 (ref. 22) (available at https://github.com/ISA-tools/ISAcreator/releases) and uploaded together with the raw data to the metabolomics repository MetaboLights^6^ (Data Citation 1). Profiles of positive mode were used for the data analyses as many important and known secondary metabolites classes in bryophytes such as flavonoids, phenylpropanoids, anthocyans, glycosides and previously characterized compounds such as Marchantins, Communins and Ohioensins ionize well in positive mode with our instrumental setup.

### Peak detection

Chromatographic peak picking was performed in R 3.4.2 (available at https://cran.r-project.org) with the package XCMS 1.52.0 (ref. 23) using the centWave algorithm and the following parameters: ppm=35, peakwidth=4,21, snthresh=10, prefilter=5–50, fitgauss=TRUE, verbose.columns=TRUE. Grouping of chromatographic peaks was performed with two factors (in XCMS called “phenoData”): *seasons* with the levels summer, autumn, winter and spring; and *species* with the levels Brarut, Calcus, Fistax, Gripul, Hypcup, Marpol, Plaund, Polstr and Rhysqu. The following parameters were used for grouping: mzwid=0.01, minfrac=0.5, bw=4. To improve subsequent data analyses, intensities in the peak table were log transformed before grouping. For further analysis, only features between the retention times 20 s and 1020 s were kept. Retention time correction was performed using the function retcor in XCMS using the parameters method=loess, family=gaussian, missing=10, extra=1, span=2. The parameters were additionally optimized using the R package IPO 1.3.3 (ref. 24), but better alignment precision was achieved with manual control and knowledge of instrument settings^25^.

### Peak annotation

Adduct annotation was performed with the R package CAMERA 1.33.3 (ref. 26) by using the following functions: xsAnnotate, groupFWHM, findIsotopes, groupCorr, findAdducts; with the following parameters: perfwhm=0.6, ppm=5, mzabs=0.005, calcIso=TRUE, calcCiS=TRUE, calcCaS=TRUE, graphMethod=lpc, pval=0.05, cor_eic_th=0.75. In order to improve subsequent statistical analyses instead of the CAMERA function getPeaklist the function getReducedPeaklist was written that aggregates the adducts of putative compounds into a feature list with singular components (see pull request in GitHub: https://github.com/sneumann/CAMERA/pull/16). Since version 1.33.3 the function getReducedPeaklist is officially part of CAMERA. The parameter method=median was chosen for the study.

### Exemplary compound annotation

Compounds were putatively annotated for the follow-up validation and biochemical interpretation with the software Bruker Compass IsotopePattern 4.4. Annotation was performed by calculating accurate masses (mass-to-charge values) from known compounds in *M. polymorpha* and other liverworts found in PubChem, the KNApSAcK database and Asakawa *et al.*^27,28^. In the software Bruker Compass DataAnalysis 4.4 the mass-to-charge was matched to device-specific retention times in the metabolite profile. To validate whether the known compound was present in the profile, Extracted Ion Chromatograms (EIC) and area-under-curve (integrated intensities) were checked manually.

### Diversity analysis

Statistical analyses were performed using the additional R packages: multtest, RColorBrewer, vegan, multcomp, multtest, nlme, ape, pvclust, dendextend, phangorn, Hmisc, gplots and VennDiagram. A presence-absence matrix was generated from the feature matrix to determine the differences in metabolite features between the experimental factors species and season. In accordance with the minfrac parameter in the alignment step in XCMS (see above), a feature was considered present when it was detected at least in two out of three replicates. The presence-absence matrix was used for measuring the metabolite richness for each species and season by calculating the Shannon diversity index (H’) for each sample *i* using the function diversity in vegan with the parameter index=shannon^29^. The following equation was used for calculation:

$$H\prime =\sum_{i=1}^{t}pi\mathrm{In}\left(pi\right)$$

where t represents the number of samples in the particular group.

The total number of features and the number of unique features were calculated from the presence-absence matrix accordingly. To test factor levels for significant differences, the Tukey HSD on a one-way ANOVA was performed post-hoc using the multcomp package.

Variability was calculated with the Pearson Correlation Coefficient (PCC, Pearson’s r) using the function rcorr in the package Hmisc. Venn diagrams were created for each species separately using the package VennDiagram. Each set in the Venn diagram represents one season and shows distinct and shared features in all possible combinations between the sets.

### Multivariate statistical analysis

Variation partitioning was performed using the function varpart in the package vegan to analyze the influence of the factors species and seasons on the metabolite profiles. Distance-based redundancy analysis (dbRDA) using the function capscale with Bray-Curtis distance and multidimensional scaling in the package vegan was chosen to analyse the relation of the ecological characteristics with the species metabolite profiles^30,31^. Ordinal and categorical ecological characteristics were transformed to presence-absence matrices for the ordination. The optimal model for the dbRDA was chosen with forward and backward selection using the function ordistep in the package vegan. Ecological characteristics were added to the plots as post-hoc variables using the function envfit in the package vegan.

### Chemotaxonomic comparison to phylogeny

Relationships between metabolite profiles and phylogeny were analysed by calculating dissimilarities for phylogeny and the feature matrix using Bray-Curtis distance (function vegdist in vegan) followed by hierarchical clustering using the function hclust and the complete linkage method. In order to improve the visual comparison between the two trees, the chemotaxonomic plot was reordered using the function order.optimal (package cba) and leaves of Polstr and Plaund were swapped using the function reorder in vegan. The similarity of the two trees was determined with the normalized Robinson-Foulds metric (function RF.dist in package phangorn). The similarity of the distance matrices was determined with the Mantel statistics (function mantel in vegan).

### Computational workflow

For the computational workflow, the required software tools, their dependencies, as well as software libraries and R packages were containerized using Docker technology^32^. The container was based on Linux and Ubuntu 16.04 and included R version 3.4.2 from the R apt repository. The commands for building the container can be found in the Dockerfile (Table 1 (available online only)). The resulting container image was made available at DockerHub (https://hub.docker.com/r/korseby/mtbls520/).

The computational workflow was constructed with the Galaxy workflow management system^33^. It consists of 20 modules and each individual module represents one or more dedicated steps in the Peters *et al.* study^9^, e.g. data retrieval, feature detection, alignment or statistical analysis (Fig. 1). For the workflow, individual Galaxy modules were written in XML format. Each Galaxy module executes a shell or R script with defined inputs and outputs. Scripts are only executed inside the software container. Thus, code execution is encapsulated and all required software dependencies were resolved in the software container. In order to comply with the *Interoperability* criterion in the FAIR guidelines^34^, the PhenoMeNal cloud e-infrastructure was used to test the workflow in different computational environments (https://phenomenal-h2020.eu). To ensure that the workflow generates the same results in different computational environments, continuous automatic workflow testing was implemented with wft4galaxy^35^.

## Data Records

The primary access site for the dataset is MetaboLights (Data Citation 1), which includes the 108 metabolite profles of the bryophytes in positive and negative mode, QC profiles, ecological data and meta-data (see Table 2 (available online only) for an overview of sample names and associated factor levels). Table 1 (available online only) provides an overview of data files, formats and functions in the computational workflow.

### Code Availability

The source code (also deposited at https://github.com/korseby/container-mtbls520/) was published^36^ and made available under the terms of APACHE license 2.0. Please refer Table 1 (available online only) for an overview of the function of each file of the source code.

Code for building the software container image and the workflow including Galaxy modules and scripts that are executed inside the container were published under Open Access^36^. A pre-built binary software container image was made available at DockerHub (retrievable at https://hub.docker.com/r/korseby/mtbls520/).

## Technical Validation

### Quality control

Four sets of 27 bryophyte samples were generated in the experiment. One set for each season was analyzed with UPLC/ESI-QTOF-MS (see methods below) which resulted in a total of 108 bryophyte metabolite profiles. In order to validate the instrument performance and to detect batch effects between the four instrument runs, a quality control (QC) protocol was implemented. Sets of 27 species samples were interspersed by samples of a lab-internal standard mix (MM8) before and after 7 bryophyte samples. Peak detection in these MM8 profiles was performed with the identical parameters as for the bryophyte samples.

**Figure 2:**
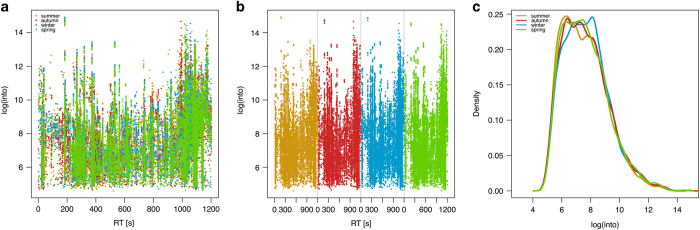
Plots of sets of the MM8 profiles to assess the performance of the technical setup. Green=spring, yellow=summer, red=autumn, blue=winter. n=28. (**a**) Plot of the four sets of MM8 profiles against each other. X axis: Retention time [s]. Y axis: Logarithmic total ion current. (**b**) Stacked plot of the sets of MM8 profiles next to each other. X axis: Retention time [s]. Y axis: Logarithmic total ion current. (**c**) Density plot (histogram) of log intensities of the sets of MM8 profiles. X axis: Sample size. Y axis: Estimated kernel density.

The four sets containing the MM8 metabolite profiles were checked visually for differences by plotting them against each other (Fig. 2a) and stacked next to each other (Fig. 2b). The density distribution of the intensities within the sets of MM8 profiles were also checked and compared to each other with a density plot (histogram) (Fig. 2c).

**Figure 3:**
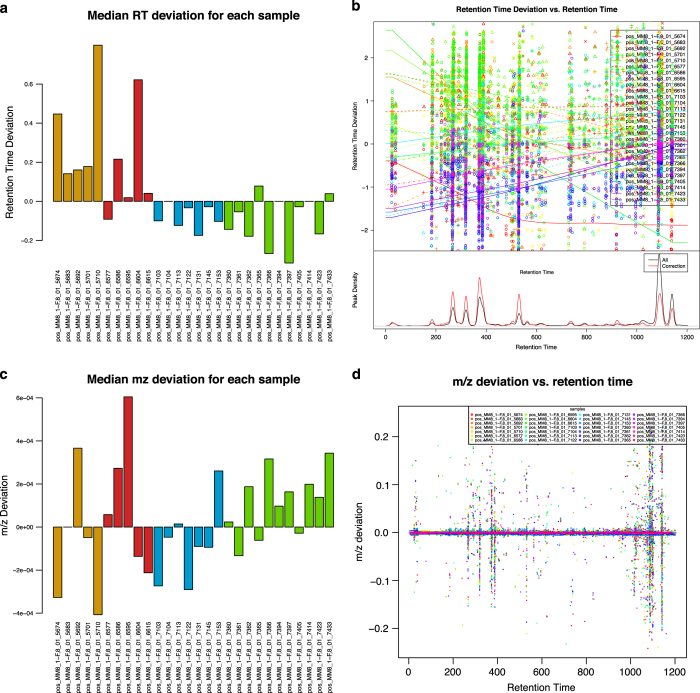
Quality control plots to assess shifts in retention time (RT) and mass-to-charge ratio (m/z) in the four sets of MM8 profiles. Green=spring, yellow=summer, red=autumn, blue=winter. n=28. (**a**) Median retention time deviation for the sets of MM8 profiles. X axis: Name of MM8 profile. Y axis: Retention time deviation [s]. (**b**) Retention time deviation plotted against retention time. X axis: Retention time [s]. Y axis: Retention time deviation [s] per profile. (**c**) Median mass-to-charge deviation for each profile. X axis: MM8 profile. Y axis: m/z deviation. (**d**) Mass-to-charge deviation plotted against retention time. X axis: Retention time [s]. Y axis: m/z deviation per profile.

Mass-to-charge ratio and retention time deviation (in seconds) and correction made by XCMS were checked with diagnostic plots made by XCMS (Fig. 3). We found maximum retention time deviations within 2 s (Fig. 3a and b) which are in the expected range of the analytical setup^18^. The determined mass-to-charge deviations (Fig. 3c and d) are within instrument specification as well^18^.

**Figure 4:**
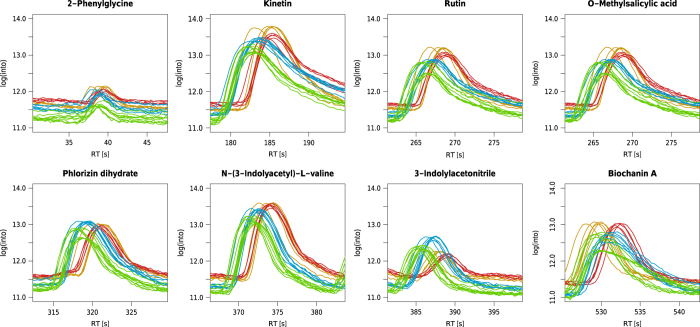
The eight compounds used for the internal lab standard mix (MM8) plotted next to each other. Shown are the regions of the respective compounds in the raw chromatograms before the alignment of XCMS. Green=spring, yellow=summer, red=autumn, blue=winter. X axis: Retention time [s]. Y axis: Logarithmic total ion current. n=28.

**Figure 5:**
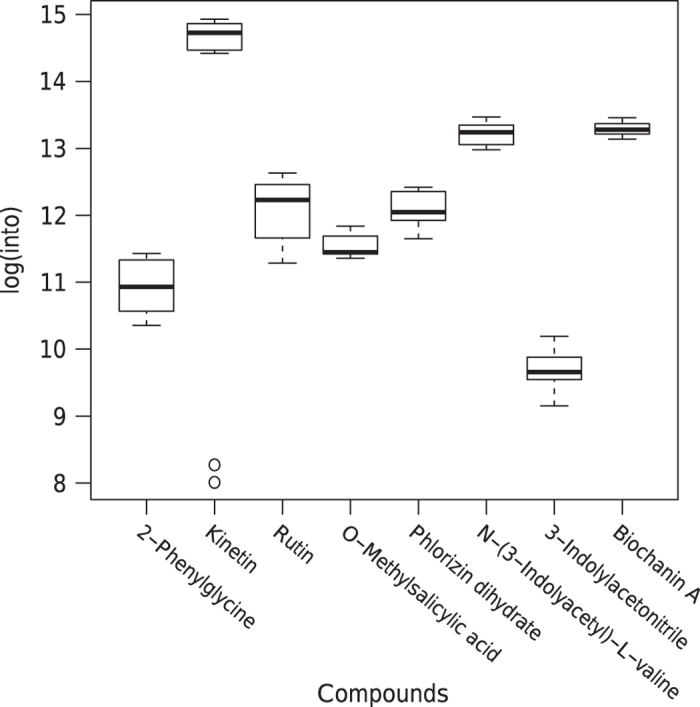
Boxplots of the variation in the intensities of the eight compounds used in the internal lab standard mix of all the MM8 profiles. X axis: Compound. Y axis: Logarithmic total ion current. n=28 for each box.

The variation in the intensities of the internal lab standards was also checked for each reference compound individually as shown in Figs. 4 and 5. In general, the variation for each reference compound and the deviations between MM8 profiles are both well within the typical range of 10 to 15% (ref. 18).

We conclude that there are no significant batch effects in the technical replicates to overlap with the factor *seasons* of the experiment. Thus, the automatic retention time correction made by XCMS is validated for the parameters used in the peak detection process.

### Exemplary annotation of *Marchantia polymorpha* profile

**Figure 6:**
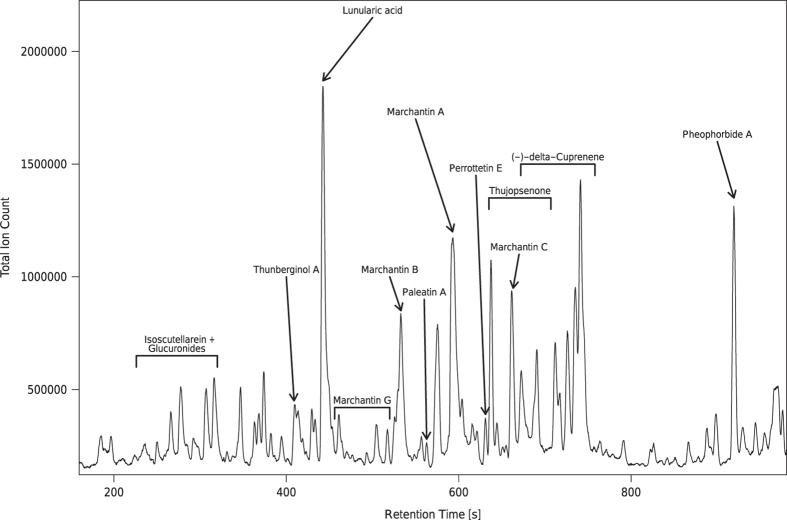
Total Ion Count (TIC) chromatogram obtained from the extracts of *Marchantia polymorpha*. This exemplary chromatogram was obtained from the third sample of summer. Values of the retention times (RT values), accurate masses and sum formulas are available in Table 3 (available online only).

With known accurate masses (m/z values) and calculated retention time values (see methods), we confirm the annotation of many known compounds which are described in literature for the model species *Marchantia polymorpha*^27,28^ (Fig. 6). Many of these known compounds also constitute the most abundant features in the profile of *M. polymorpha* (Fig. 6).

### Computational workflow

We have implemented the computational workflow in the Galaxy workflow management system^33^ and have made the workflow and underlying code available as Open Source^36^. The Galaxy workflow represents the entire computational processing pipeline that is used in the Peters *et al.* study^9^ (Fig. 1). Each of the individual modules represents a particular step in the workflow and has defined inputs (e.g. pre-processed peak table data matrix) and outputs (e.g. PDF containing the plot of a particular statistical method) (Fig. 1). We used data standards and minimum information criteria for constructing the modules of the workflow^20,22^. Continuous automatic testing of the workflow was performed with wft4galaxy^35^ in the PhenoMeNal e-infrastructure (https://phenomenal-h2020.eu) to ensure that the workflow generates the same results in different computational environments.

We proceeded according to the FAIR guiding principles^34^ in order to implement a reusable computational workflow. The acronym FAIR stands for Findable, Accessible, Interoperable and Reusable and encompasses several criteria to support the reuse of scholarly data. So far, the FAIR guidelines have only been aspired to make data reusable. However, as the conceptual formulation within FAIR are quite generalized^37^, these principles can also be applied to computational workflows. Nonetheless, there are some computational challenges involved. For example, software runs in different software environments and software dependencies need to be resolved. We tackle this by creating software containers which can be run on multiple systems and contain the software tools, all required libraries and R packages^32,38^. As dependencies in the container have already been resolved, sharing the container image greatly facilitates to allow the software to be run in multiple environments.

We have chosen the Galaxy Workflow Management system^33,39^ to implement the whole data processing pipeline (Fig. 1) as it is already known to facilitate reproducible results^40^. Several processing modules were constructed that represent the individual steps of the Peters *et al.* study^9^. Software tools are invoked from the Galaxy modules and are executed inside the container, thus, adding a level of encapsulation and eliminating the need for the user to install additional software^41^. Galaxy has a graphical user interface that hides the technical complexity from the end user and does not need intensive bioinformatic background knowledge to run the particular modules and workflows. This greatly contributes to the adoption by the end users (biochemists and ecologists) and facilitates future studies in the research field of Eco-Metabolomics.

### Statistical analyses

With untargeted metabolomics analysis in ecology, diversity analysis is typically used to characterize the richness and the abundance of biochemical features in the metabolite profiles of biological species^42^. Metabolite richness is a simple measure that counts the individual biochemical features in the metabolite profiles of the species^43^. The abundance of features in the metabolite profiles is usually calculated by diversity indices such as the Shannon diversity index (H’) in order to characterize simple relationships with regard to the study factors^44^.

Ordination methods such as Redundancy Analysis (RDA) and distance-based Redundancy Analysis (dbRDA) are frequently used in Ecology^30^. They allow to derive correlations of specific variables between the matrix of predictors containing the measurements (X matrix) and the response matrix with the ecological traits (Y matrix)^30,45^. These methods are also suitable for Eco-Metabolomics data as they allow the use of multiple (non-categorial) variables in a single model and allow to calculate the amount of explained variance of the model. We have chosen the dbRDA, which can also be regarded as a constrained version of metric scaling (MDS)^46,47^. We have implemented dedicated modules for these statistical operations in our computational workflow (see Methods section and Fig. 1).

## Usage Notes

### Building the container image

Following are instructions to manually build the container image. The file Dockerfile in Table 1 (available online only) contains the ruleset. The container has been built using Docker version 17.05-ce under Linux Ubuntu 16.04. The following commands were run to generate the image:

sudo apt-get install apt-transport-https ca-certificates git sudo echo “deb http://apt.dockerproject.org/repo ubuntu-xenial main” >>/etc/apt/sources.list sudo apt-key adv --keyserver hkp://ha.pool.sks-keyservers.net :80 --recv-keys 58118E89F3A912897C070ADBF76221572C52609D sudo apt-get update && sudo apt-get install docker git clone https://github.com/korseby/container-mtbls520 cd container-mtbls520 docker build -t korseby/mtbls520.

### Installing and using Galaxy to run the workflow

The workflow was tested with Galaxy version 17.09. Instructions how to install Galaxy can be found in the training material of the Galaxy project (accessible at https://galaxyproject.github.io/training-material/). However, it is recommended that an official Galaxy server is used, such as those from the PhenoMeNal infrastructure (available at https://public.phenomenal-h2020.eu/).

After being logged into Galaxy, a click on “Workflow” in the menu bar on the top and then a click on the “Upload” button opens up a new page. In the field “Galaxy workflow URL:” enter the following address “https://raw.githubusercontent.com/korseby/container-mtbls520/develop/galaxy/mtbls520_workflow.ga” or upload the .ga file from the GitHub repository (Table 1 (available online only)) and then clicking on the button “Import”. This will import the workflow of the study into Galaxy. The workflow will now be available in Galaxy under Workflows as “Metabolights 520 Eco-Metabolomics Workflow”. From there, clicking on the drop-down menu there are options to “Edit” (visually view the complete workflow in the Galaxy workflow editor) or to “Run” the workflow. Required data can be downloaded from MetaboLights with the Galaxy module “mtbls520_01_mtbls_download” (Table 1 (available online only)). Once the download has been completed, data can be extracted with the Galaxy module “mtbls520_02_extract” (Table 1 (available online only)). The workflow can be directly run once the inputs have been assigned to the extracted data files. Processing will take approx. 40 min depending on the work load of the computational infrastructure.

## Additional information

**How to cite this article**: Peters, K. *et al*. Computational workflow to study the seasonal variation of secondary metabolites in nine different bryophytes. *Sci. Data* 5:180179 doi: 10.1038/sdata.2018.179 (2018).

**Publisher’s note**: Springer Nature remains neutral with regard to jurisdictional claims in published maps and institutional affiliations.

## Supplementary Material



## Figures and Tables

**Table 1:** Names, types, descriptions and locations of primary data and additional scripts referenced in this article

File Name	Description	Type	Location	URL	Data Record Accession
Dockerfile	Source code for building the container image	Text	GitHub	https://github.com/korseby/container-mtbls520	korseby/container-mtbls520
galaxy/mtbls520_workflow.ga	Galaxy workflow	XML	GitHub	https://github.com/korseby/container-mtbls520	korseby/container-mtbls520
galaxy/mtbls520_workflow.jpg	Screenshot of the workflow running in Galaxy	JPG	GitHub	https://github.com/korseby/container-mtbls520	korseby/container-mtbls520
korseby/mtbls520	Container image	Container	Dockerhub	https://hub.docker.com/r/korseby/mtbls520/	korseby/mtbls520
m_bryos_metabolite_*_negative_mode.maf	Peak table matrix of bryophyte samples (negative mode)	Tabular Separated	MetaboLights 520 (Data Citation 1)	https://www.ebi.ac.uk/metabolights/MTBLS520	MTBLS520
m_bryos_metabolite_*_positive_mode.maf	Peak table matrix of bryophyte samples (positive mode)	Tabular Separated	MetaboLights 520 (Data Citation 1)	https://www.ebi.ac.uk/metabolights/MTBLS520	MTBLS520
m_bryos_quality_*_negative_mode.maf	Peak table matrix of QC samples (negative mode)	Tabular Separated	MetaboLights 520 (Data Citation 1)	https://www.ebi.ac.uk/metabolights/MTBLS520	MTBLS520
m_bryos_quality_*_positive_mode.maf	Peak table matrix of QC samples (positivemode)	Tabular Separated	MetaboLights 520 (Data Citation 1)	https://www.ebi.ac.uk/metabolights/MTBLS520	MTBLS520
m_characteristics.csv	Ecological characteristics of the bryophyte species compiled from literature	Comma Separated	MetaboLights 520 (Data Citation 1)	https://www.ebi.ac.uk/metabolights/MTBLS520	MTBLS520, doi:<NEW_PHYTOL>
m_moss_phylo.tre	Phylogenetic distances of bryophyte species	Tree	MetaboLights 520 (Data Citation 1)	https://www.ebi.ac.uk/metabolights/MTBLS520	MTBLS520
mtbls520_01_mtbls_download.sh	Script: Download the whole study from MetaboLights	Shell script	GitHub	https://github.com/korseby/container-mtbls520	korseby/container-mtbls520
mtbls520_01_mtbls_download.xml	Galaxy module: Download the whole study from MetaboLights	XML	GitHub	https://github.com/korseby/container-mtbls520	korseby/container-mtbls520
mtbls520_02_extract.sh	Script: Extract files from a downloaded MetaboLights archive	Shell script	GitHub	https://github.com/korseby/container-mtbls520	korseby/container-mtbls520
mtbls520_02_extract.xml	Galaxy module: Extract files from the downloaded MetaboLights archive	XML	GitHub	https://github.com/korseby/container-mtbls520	korseby/container-mtbls520
mtbls520_03_qc_perform.r	Script: Perform Quality Control	R script	GitHub	https://github.com/korseby/container-mtbls520	korseby/container-mtbls520
mtbls520_03_qc_preparations.sh	Script: Make preparations for the QC	Shell script	GitHub	https://github.com/korseby/container-mtbls520	korseby/container-mtbls520
mtbls520_03_quality_control.xml	Galaxy module: Process the quality control pipeline	XML	GitHub	https://github.com/korseby/container-mtbls520	korseby/container-mtbls520
mtbls520_04_preparations.r	Script: Preparations and settings for files and the R environment	R script	GitHub	https://github.com/korseby/container-mtbls520	korseby/container-mtbls520
mtbls520_04_preparations.sh	Script: Import peak table matrix	Shell script	GitHub	https://github.com/korseby/container-mtbls520	korseby/container-mtbls520
mtbls520_04_preparations.xml	Galaxy module: Preparations and settings for files and the R environment	XML	GitHub	https://github.com/korseby/container-mtbls520	korseby/container-mtbls520
mtbls520_05a_import_maf.r	Script: Generating matrix for diversity calculations	R script	GitHub	https://github.com/korseby/container-mtbls520	korseby/container-mtbls520
mtbls520_05a_import_maf.xml	Galaxy module: Import peak table matrix	XML	GitHub	https://github.com/korseby/container-mtbls520	korseby/container-mtbls520
mtbls520_06_import_traits.r	Script: Import ecological characteristics	R script	GitHub	https://github.com/korseby/container-mtbls520	korseby/container-mtbls520
mtbls520_06_import_traits.xml	Galaxy module: Import ecological characteristics	XML	GitHub	https://github.com/korseby/container-mtbls520	korseby/container-mtbls520
mtbls520_07_species_diversity.r	Script: Create species unique features plot	R script	GitHub	https://github.com/korseby/container-mtbls520	korseby/container-mtbls520
mtbls520_07_species_diversity.xml	Galaxy module: Generating matrix for diversity calculations	XML	GitHub	https://github.com/korseby/container-mtbls520	korseby/container-mtbls520
mtbls520_08a_species_shannon.r	Script: Create species Shannon diversity plot	R script	GitHub	https://github.com/korseby/container-mtbls520	korseby/container-mtbls520
mtbls520_08a_species_shannon.xml	Galaxy module: Create species Shannon diversity plot	XML	GitHub	https://github.com/korseby/container-mtbls520	korseby/container-mtbls520
mtbls520_08b_species_unique.r	Script: Create species unique features plot	R script	GitHub	https://github.com/korseby/container-mtbls520	korseby/container-mtbls520
mtbls520_08b_species_unique.xml	Galaxy module: Create species unique features plot	XML	GitHub	https://github.com/korseby/container-mtbls520	korseby/container-mtbls520
mtbls520_08c_species_variability.r	Script: Create species variability plot	R script	GitHub	https://github.com/korseby/container-mtbls520	korseby/container-mtbls520
mtbls520_08c_species_variability.xml	Galaxy module: Create species variability plot	XML	GitHub	https://github.com/korseby/container-mtbls520	korseby/container-mtbls520
mtbls520_08d_concentration.xml	Galaxy module: Create species metabolite concentration plot	XML	GitHub	https://github.com/korseby/container-mtbls520	korseby/container-mtbls520
mtbls520_08d_species_concentration.r	Script: Create species concentration plot	R script	GitHub	https://github.com/korseby/container-mtbls520	korseby/container-mtbls520
mtbls520_09_species_venn.r	Script: Create Venn diagram plots	R script	GitHub	https://github.com/korseby/container-mtbls520	korseby/container-mtbls520
mtbls520_09_species_venn.xml	Galaxy module: Create species Venn plots	XML	GitHub	https://github.com/korseby/container-mtbls520	korseby/container-mtbls520
mtbls520_10_species_varpart.r	Script: Create variation partitioning plot	R script	GitHub	https://github.com/korseby/container-mtbls520	korseby/container-mtbls520
mtbls520_10_species_varpart.xml	Galaxy module: Create variation partitioning plot	XML	GitHub	https://github.com/korseby/container-mtbls520	korseby/container-mtbls520
mtbls520_12_species_marchantia.r	Script: Create annotation of Marchantia polymorpha profile	R script	GitHub	https://github.com/korseby/container-mtbls520	korseby/container-mtbls520
mtbls520_12_species_marchantia.xml	Galaxy module: Create annotation of Marchantia polymorpha profile plot	XML	GitHub	https://github.com/korseby/container-mtbls520	korseby/container-mtbls520
mtbls520_16_ecology_rda.r	Script: Create ecology dbRDA plot	R script	GitHub	https://github.com/korseby/container-mtbls520	korseby/container-mtbls520
mtbls520_16_ecology_rda.xml	Galaxy module: Create ecology dbRDA plot	XML	GitHub	https://github.com/korseby/container-mtbls520	korseby/container-mtbls520
mtbls520_18_phylogeny.r	Script: Create phylogeny and chemotaxonomy plot	R script	GitHub	https://github.com/korseby/container-mtbls520	korseby/container-mtbls520
mtbls520_18_phylogeny.xml	Galaxy module: Create phylogeny and chemotaxonomy plot	XML	GitHub	https://github.com/korseby/container-mtbls520	korseby/container-mtbls520
mtbls520_19a_seasons_shannon.r	Script: Create seasons Shannon diversity plot	R script	GitHub	https://github.com/korseby/container-mtbls520	korseby/container-mtbls520
mtbls520_19a_seasons_shannon.xml	Galaxy module: Create seasons Shannon diversity plot	XML	GitHub	https://github.com/korseby/container-mtbls520	korseby/container-mtbls520
mtbls520_19b_seasons_unique.r	Script: Create seasons unique features plot	R script	GitHub	https://github.com/korseby/container-mtbls520	korseby/container-mtbls520
mtbls520_19b_seasons_unique.xml	Galaxy module: Create seasons unique features plot	XML	GitHub	https://github.com/korseby/container-mtbls520	korseby/container-mtbls520
mtbls520_19c_seasons_variability.r	Script: Create seasons variability plot	R script	GitHub	https://github.com/korseby/container-mtbls520	korseby/container-mtbls520
mtbls520_19c_seasons_variability.xml	Galaxy module: Create seasons variability plot	XML	GitHub	https://github.com/korseby/container-mtbls520	korseby/container-mtbls520
mtbls520_19d_seasons_concentration.r	Script: Create seasons concentration plot	R script	GitHub	https://github.com/korseby/container-mtbls520	korseby/container-mtbls520
mtbls520_19d_seasons_concentration.xml	Galaxy module: Create seasons metabolite concentration plot	XML	GitHub	https://github.com/korseby/container-mtbls520	korseby/container-mtbls520
mtbls520_23_seasons_rda.r	Script: Create seasons dbRDA plot	R script	GitHub	https://github.com/korseby/container-mtbls520	korseby/container-mtbls520
mtbls520_23_seasons_rda.xml	Galaxy module: Create seasons RDA plot	XML	GitHub	https://github.com/korseby/container-mtbls520	korseby/container-mtbls520
neg_[01-27]_*.mzML	Metabolite profiles of the bryophyte species measured in negative mode	Raw	MetaboLights 520 (Data Citation 1)	https://www.ebi.ac.uk/metabolights/MTBLS520	MTBLS520
neg_MM8_*.mzML	Quality control profiles measured in negative mode	Raw	MetaboLights 520 (Data Citation 1)	https://www.ebi.ac.uk/metabolights/MTBLS520	MTBLS520
pos_[01-27]_*.mzML	Metabolite profiles of the bryophyte species measured in positive mode	Raw	MetaboLights 520 (Data Citation 1)	https://www.ebi.ac.uk/metabolights/MTBLS520	MTBLS520
pos_MM8_*.mzML	Quality control profiles measured in positive mode	Raw	MetaboLights 520 (Data Citation 1)	https://www.ebi.ac.uk/metabolights/MTBLS520	MTBLS520

**Table 2:** Overview of sample names and associated factor levels of the study

Sample Name	Type	Species	Season	Replicate
pos_01_Fistax_1-A.2_01_5675	Bryophyte species metabolite profile	Fissidens taxifolius	Summer	01
pos_02_Fistax_1-A.3_01_5676	Bryophyte species metabolite profile	Fissidens taxifolius	Summer	02
pos_03_Fistax_1-A.4_01_5677	Bryophyte species metabolite profile	Fissidens taxifolius	Summer	03
pos_04_Polstr_1-A.5_01_5678	Bryophyte species metabolite profile	Polytrichum strictum	Summer	01
pos_05_Polstr_1-A.6_01_5679	Bryophyte species metabolite profile	Polytrichum strictum	Summer	02
pos_06_Polstr_1-A.7_01_5680	Bryophyte species metabolite profile	Polytrichum strictum	Summer	03
pos_07_Hypcup_1-A.8_01_5681	Bryophyte species metabolite profile	Hypnum cupressiforme	Summer	01
pos_08_Gripul_1-B.1_01_5684	Bryophyte species metabolite profile	Grimmia pulvinata	Summer	01
pos_09_Plaund_1-B.2_01_5685	Bryophyte species metabolite profile	Plagiomnium undulatum	Summer	01
pos_10_Plaund_1-B.3_01_5686	Bryophyte species metabolite profile	Plagiomnium undulatum	Summer	02
pos_11_Plaund_1-B.4_01_5687	Bryophyte species metabolite profile	Plagiomnium undulatum	Summer	03
pos_12_Rhysqu_1-B.5_01_5688	Bryophyte species metabolite profile	Rhytidiadelphus squarrosus	Summer	01
pos_13_Rhysqu_1-B.6_01_5689	Bryophyte species metabolite profile	Rhytidiadelphus squarrosus	Summer	02
pos_14_Calcus_1-B.7_01_5690	Bryophyte species metabolite profile	Calliergonella cuspidata	Summer	01
pos_15_Calcus_1-C.1_01_5693	Bryophyte species metabolite profile	Calliergonella cuspidata	Summer	02
pos_16_Rhysqu_1-C.2_01_5694	Bryophyte species metabolite profile	Rhytidiadelphus squarrosus	Summer	03
pos_17_Calcus_1-C.3_01_5695	Bryophyte species metabolite profile	Calliergonella cuspidata	Summer	03
pos_18_Brarut_1-C.4_01_5696	Bryophyte species metabolite profile	Brachythecium rutabulum	Summer	01
pos_19_Brarut_1-C.5_01_5697	Bryophyte species metabolite profile	Brachythecium rutabulum	Summer	02
pos_20_Hypcup_1-C.6_01_5698	Bryophyte species metabolite profile	Hypnum cupressiforme	Summer	02
pos_21_Hypcup_1-C.7_01_5699	Bryophyte species metabolite profile	Hypnum cupressiforme	Summer	03
pos_22_Gripul_1-D.1_01_5702	Bryophyte species metabolite profile	Grimmia pulvinata	Summer	02
pos_23_Gripul_1-D.2_01_5703	Bryophyte species metabolite profile	Grimmia pulvinata	Summer	03
pos_24_Brarut_1-D.3_01_5704	Bryophyte species metabolite profile	Brachythecium rutabulum	Summer	03
pos_25_Marpol_1-D.4_01_5705	Bryophyte species metabolite profile	Marchantia polymorpha	Summer	01
pos_26_Marpol_1-D.5_01_5706	Bryophyte species metabolite profile	Marchantia polymorpha	Summer	02
pos_27_Marpol_1-D.6_01_5707	Bryophyte species metabolite profile	Marchantia polymorpha	Summer	03
pos_01_Fistax_1-A.2_01_6578	Bryophyte species metabolite profile	Fissidens taxifolius	Autumn	01
pos_02_Fistax_1-A.3_01_6579	Bryophyte species metabolite profile	Fissidens taxifolius	Autumn	02
pos_03_Fistax_1-A.4_01_6580	Bryophyte species metabolite profile	Fissidens taxifolius	Autumn	03
pos_04_Hypcup_1-A.5_01_6581	Bryophyte species metabolite profile	Hypnum cupressiforme	Autumn	01
pos_05_Gripul_1-A.6_01_6582	Bryophyte species metabolite profile	Grimmia pulvinata	Autumn	01
pos_06_Brarut_1-A.7_01_6583	Bryophyte species metabolite profile	Brachythecium rutabulum	Autumn	01
pos_07_Polstr_1-A.8_01_6584	Bryophyte species metabolite profile	Polytrichum strictum	Autumn	01
pos_08_Polstr_1-B.1_01_6587	Bryophyte species metabolite profile	Polytrichum strictum	Autumn	02
pos_09_Polstr_1-B.2_01_6588	Bryophyte species metabolite profile	Polytrichum strictum	Autumn	03
pos_10_Hypcup_1-B.3_01_6589	Bryophyte species metabolite profile	Hypnum cupressiforme	Autumn	02
pos_11_Gripul_1-B.4_01_6590	Bryophyte species metabolite profile	Grimmia pulvinata	Autumn	02
pos_12_Brarut_1-B.5_01_6591	Bryophyte species metabolite profile	Brachythecium rutabulum	Autumn	02
pos_13_Plaund_1-B.6_01_6592	Bryophyte species metabolite profile	Plagiomnium undulatum	Autumn	01
pos_14_Plaund_1-B.7_01_6593	Bryophyte species metabolite profile	Plagiomnium undulatum	Autumn	02
pos_15_Plaund_1-C.1_01_6596	Bryophyte species metabolite profile	Plagiomnium undulatum	Autumn	03
pos_16_Calcus_1-C.2_01_6597	Bryophyte species metabolite profile	Calliergonella cuspidata	Autumn	01
pos_17_Calcus_1-C.3_01_6598	Bryophyte species metabolite profile	Calliergonella cuspidata	Autumn	02
pos_18_Rhysqu_1-C.4_01_6599	Bryophyte species metabolite profile	Rhytidiadelphus squarrosus	Autumn	01
pos_19_Rhysqu_1-C.5_01_6600	Bryophyte species metabolite profile	Rhytidiadelphus squarrosus	Autumn	02
pos_20_Calcus_1-C.6_01_6601	Bryophyte species metabolite profile	Calliergonella cuspidata	Autumn	03
pos_21_Rhysqu_1-C.7_01_6602	Bryophyte species metabolite profile	Rhytidiadelphus squarrosus	Autumn	03
pos_22_Hypcup_1-D.1_01_6605	Bryophyte species metabolite profile	Hypnum cupressiforme	Autumn	03
pos_23_Gripul_1-D.2_01_6609	Bryophyte species metabolite profile	Grimmia pulvinata	Autumn	03
pos_24_Brarut_1-D.3_01_6610	Bryophyte species metabolite profile	Brachythecium rutabulum	Autumn	03
pos_25_Marpol_1-D.4_01_6611	Bryophyte species metabolite profile	Marchantia polymorpha	Autumn	01
pos_26_Marpol_1-D.5_01_6612	Bryophyte species metabolite profile	Marchantia polymorpha	Autumn	02
pos_27_Marpol_1-D.6_01_6613	Bryophyte species metabolite profile	Marchantia polymorpha	Autumn	03
pos_01_Fistax_1-A.2_01_7105	Bryophyte species metabolite profile	Fissidens taxifolius	Winter	01
pos_02_Fistax_1-A.3_01_7106	Bryophyte species metabolite profile	Fissidens taxifolius	Winter	02
pos_03_Fistax_1-A.4_01_7107	Bryophyte species metabolite profile	Fissidens taxifolius	Winter	03
pos_04_Hypcup_1-A.5_01_7108	Bryophyte species metabolite profile	Hypnum cupressiforme	Winter	01
pos_05_Brarut_1-A.6_01_7109	Bryophyte species metabolite profile	Brachythecium rutabulum	Winter	01
pos_06_Polstr_1-A.7_01_7110	Bryophyte species metabolite profile	Polytrichum strictum	Winter	01
pos_07_Polstr_1-A.8_01_7111	Bryophyte species metabolite profile	Polytrichum strictum	Winter	02
pos_08_Polstr_1-A.1_01_7114	Bryophyte species metabolite profile	Polytrichum strictum	Winter	03
pos_09_Gripul_1-A.2_01_7115	Bryophyte species metabolite profile	Grimmia pulvinata	Winter	01
pos_10_Hypcup_1-A.3_01_7116	Bryophyte species metabolite profile	Hypnum cupressiforme	Winter	02
pos_11_Plaund_1-A.4_01_7117	Bryophyte species metabolite profile	Plagiomnium undulatum	Winter	01
pos_12_Plaund_1-A.5_01_7118	Bryophyte species metabolite profile	Plagiomnium undulatum	Winter	02
pos_13_Plaund_1-A.6_01_7119	Bryophyte species metabolite profile	Plagiomnium undulatum	Winter	03
pos_14_Rhysqu_1-A.7_01_7120	Bryophyte species metabolite profile	Rhytidiadelphus squarrosus	Winter	01
pos_15_Rhysqu_1-A.8_01_7138	Bryophyte species metabolite profile	Rhytidiadelphus squarrosus	Winter	02
pos_16_Rhysqu_1-A.1_01_7139	Bryophyte species metabolite profile	Rhytidiadelphus squarrosus	Winter	03
pos_17_Calcus_1-A.2_01_7140	Bryophyte species metabolite profile	Calliergonella cuspidata	Winter	01
pos_18_Calcus_1-A.3_01_7141	Bryophyte species metabolite profile	Calliergonella cuspidata	Winter	02
pos_19_Calcus_1-A.4_01_7142	Bryophyte species metabolite profile	Calliergonella cuspidata	Winter	03
pos_20_Brarut_1-A.5_01_7143	Bryophyte species metabolite profile	Brachythecium rutabulum	Winter	02
pos_21_Hypcup_1-A.6_01_7144	Bryophyte species metabolite profile	Hypnum cupressiforme	Winter	03
pos_22_Brarut_1-A.7_01_7146	Bryophyte species metabolite profile	Brachythecium rutabulum	Winter	03
pos_23_Gripul_1-A.8_01_7147	Bryophyte species metabolite profile	Grimmia pulvinata	Winter	02
pos_24_Gripul_1-A.1_01_7148	Bryophyte species metabolite profile	Grimmia pulvinata	Winter	03
pos_25_Marpol_1-A.2_01_7149	Bryophyte species metabolite profile	Marchantia polymorpha	Winter	01
pos_26_Marpol_1-A.3_01_7150	Bryophyte species metabolite profile	Marchantia polymorpha	Winter	02
pos_27_Marpol_1-A.4_01_7151	Bryophyte species metabolite profile	Marchantia polymorpha	Winter	03
pos_01_Fistax_1-A.2_01_7396	Bryophyte species metabolite profile	Fissidens taxifolius	Spring	01
pos_02_Fistax_1-A.3_01_7398	Bryophyte species metabolite profile	Fissidens taxifolius	Spring	02
pos_03_Fistax_1-A.4_01_7399	Bryophyte species metabolite profile	Fissidens taxifolius	Spring	03
pos_04_Gripul_1-A.5_01_7400	Bryophyte species metabolite profile	Grimmia pulvinata	Spring	01
pos_05_Brarut_1-A.6_01_7401	Bryophyte species metabolite profile	Brachythecium rutabulum	Spring	01
pos_06_Polstr_1-A.7_01_7402	Bryophyte species metabolite profile	Polytrichum strictum	Spring	01
pos_07_Polstr_1-A.8_01_7403	Bryophyte species metabolite profile	Polytrichum strictum	Spring	02
pos_08_Polstr_1-B.1_01_7406	Bryophyte species metabolite profile	Polytrichum strictum	Spring	03
pos_09_Hypcup_1-B.2_01_7407	Bryophyte species metabolite profile	Hypnum cupressiforme	Spring	01
pos_10_Brarut_1-B.3_01_7408	Bryophyte species metabolite profile	Brachythecium rutabulum	Spring	02
pos_11_Brarut_1-B.4_01_7409	Bryophyte species metabolite profile	Brachythecium rutabulum	Spring	03
pos_12_Plaund_1-B.5_01_7410	Bryophyte species metabolite profile	Plagiomnium undulatum	Spring	01
pos_13_Plaund_1-B.6_01_7411	Bryophyte species metabolite profile	Plagiomnium undulatum	Spring	02
pos_14_Plaund_1-B.7_01_7412	Bryophyte species metabolite profile	Plagiomnium undulatum	Spring	03
pos_15_Calcus_1-B.8_01_7415	Bryophyte species metabolite profile	Calliergonella cuspidata	Spring	01
pos_16_Rhysqu_1-C.1_01_7416	Bryophyte species metabolite profile	Rhytidiadelphus squarrosus	Spring	01
pos_17_Calcus_1-C.2_01_7417	Bryophyte species metabolite profile	Calliergonella cuspidata	Spring	02
pos_18_Rhysqu_1-C.3_01_7418	Bryophyte species metabolite profile	Rhytidiadelphus squarrosus	Spring	02
pos_19_Rhysqu_1-C.4_01_7419	Bryophyte species metabolite profile	Rhytidiadelphus squarrosus	Spring	03
pos_20_Calcus_1-C.5_01_7420	Bryophyte species metabolite profile	Calliergonella cuspidata	Spring	03
pos_21_Hypcup_1-C.6_01_7421	Bryophyte species metabolite profile	Hypnum cupressiforme	Spring	02
pos_22_Hypcup_1-C.7_01_7424	Bryophyte species metabolite profile	Hypnum cupressiforme	Spring	03
pos_23_Gripul_1-C.8_01_7425	Bryophyte species metabolite profile	Grimmia pulvinata	Spring	02
pos_24_Gripul_1-D.1_01_7426	Bryophyte species metabolite profile	Grimmia pulvinata	Spring	03
pos_25_Marpol_1-D.2_01_7427	Bryophyte species metabolite profile	Marchantia polymorpha	Spring	01
pos_26_Marpol_1-D.3_01_7428	Bryophyte species metabolite profile	Marchantia polymorpha	Spring	02
pos_27_Marpol_1-D.4_01_7429	Bryophyte species metabolite profile	Marchantia polymorpha	Spring	03
pos_MM8_1-F.8_01_5674	QC profile	-	summer	01
pos_MM8_1-F.8_01_5683	QC profile	-	summer	02
pos_MM8_1-F.8_01_5692	QC profile	-	summer	03
pos_MM8_1-F.8_01_5701	QC profile	-	summer	04
pos_MM8_1-F.8_01_5710	QC profile	-	summer	05
pos_MM8_1-F.8_01_6577	QC profile	-	autumn	01
pos_MM8_1-F.8_01_6586	QC profile	-	autumn	02
pos_MM8_1-F.8_01_6595	QC profile	-	autumn	03
pos_MM8_1-F.8_01_6604	QC profile	-	autumn	04
pos_MM8_1-F.8_01_6615	QC profile	-	autumn	05
pos_MM8_1-F.8_01_7103	QC profile	-	winter	01
pos_MM8_1-F.8_01_7104	QC profile	-	winter	02
pos_MM8_1-F.8_01_7113	QC profile	-	winter	03
pos_MM8_1-F.8_01_7122	QC profile	-	winter	04
pos_MM8_1-F.8_01_7131	QC profile	-	winter	05
pos_MM8_1-F.8_01_7145	QC profile	-	winter	06
pos_MM8_1-F.8_01_7153	QC profile	-	winter	07
pos_MM8_1-F.8_01_7360	QC profile	-	spring	01
pos_MM8_1-F.8_01_7361	QC profile	-	spring	02
pos_MM8_1-F.8_01_7362	QC profile	-	spring	03
pos_MM8_1-F.8_01_7365	QC profile	-	spring	04
pos_MM8_1-F.8_01_7366	QC profile	-	spring	05
pos_MM8_1-F.8_01_7394	QC profile	-	spring	06
pos_MM8_1-F.8_01_7397	QC profile	-	spring	07
pos_MM8_1-F.8_01_7405	QC profile	-	spring	08
pos_MM8_1-F.8_01_7414	QC profile	-	spring	09
pos_MM8_1-F.8_01_7423	QC profile	-	spring	10
pos_MM8_1-F.8_01_7433	QC profile	-	spring	11

**Table 3:** Some putatively annotated compounds in the metabolite profile “pos_27_Marpol_1-D.6_01_5707” of *Marchantia polymorpha* in the summer season

Compound	Sum formula	m/z	RT [s]	Area (Integrated Intensity)
(-)-delta-Cuparenol	C_15_H_22_O	219.1747	661.0	642907.19
(-)-delta-Cuparenol	C_15_H_22_O	241.1559	655.3	27828.00
(-)-delta-Cuparenol	C_15_H_22_O	241.1559	644.1	167619.28
(-)-delta-Cuparenol	C_15_H_22_O	219.1745	637.5	808057.69
(-)-delta-Cuparenol	C_15_H_22_O	219.1743	690.7	537473.44
(-)-delta-Cuparenol	C_15_H_22_O	203.1794	672.8	167255.34
(-)-delta-Cuparenol	C_15_H_22_O	219.1743	706.7	44150.91
(-)-delta-Cuprenene	C_15_H_24_	205.1952	712.0	1508130.50
(-)-delta-Cuprenene	C_15_H_25_	205.1949	764.1	63622.58
(-)-delta-Cuprenene	C_15_H_26_	205.1950	757.5	58668.16
(-)-delta-Cuprenene	C_15_H_27_	205.1949	743.7	819416.63
(-)-delta-Cuprenene	C_15_H_28_	205.1952	735.2	1419703.50
(-)-delta-Cuprenene	C_15_H_29_	205.1952	726.2	1152596.13
(-)-delta-Cuprenene	C_15_H_30_	205.1951	693.4	78010.95
(-)-delta-Cuprenene	C_15_H_31_	205.1950	687.3	318694.16
(-)-delta-Cuprenene	C_15_H_32_	205.1949	679.5	128213.59
(-)-delta-Cuprenene	C_15_H_33_	205.1949	672.0	223135.42
2,7-Dihydroxy-3-methoxyphenanthrene	C_15_H_12_O_3_	241.0859	442.5	1805637.88
2-Hydroxy-3,6-dimethoxyphenanthrene	C_16_H_14_O_3_	479.0818	266.3	6064.95
2-Hydroxy-3,6-dimethoxyphenanthrene	C_16_H_14_O_4_	463.0869	277.9	81793.41
3-Hydroxy-2,5-dimethoxyphenanthrene	C_16_H_14_O_5_	479.0818	266.3	6064.95
3-Hydroxy-2,5-dimethoxyphenanthrene	C_16_H_14_O_6_	463.0869	277.9	81793.41
8-Hydroxyluteolin 8-glucuronide	C_21_H_18_O_13_	479.0816	267.1	70267.63
Apigenin 7-galacturonide	C_21_H_18_O_11_	447.0919	298.3	77469.98
Apigenin 7-galacturonide	C_21_H_18_O_11_	447.0919	306.8	1109165.63
Aureusidin 6-glucuronide	C_21_H_18_O_12_	463.0872	318.1	695087.38
Aureusidin 6-glucuronide	C_21_H_18_O_13_	463.0862	313.2	18427.22
Aureusidin 6-glucuronide	C_21_H_18_O_14_	463.0868	304.4	22474.41
Aureusidin 6-glucuronide	C_21_H_18_O_15_	463.0865	295.0	180278.44
Aureusidin 6-glucuronide	C_21_H_18_O_16_	463.0870	277.4	1091277.38
Isoscutellarein 8-glucuronide	C_21_H_18_O_12_	463.0872	318.1	695087.38
Isoscutellarein 8-glucuronide	C_21_H_18_O_12_	463.0868	304.4	22474.41
Isoscutellarein 8-glucuronide	C_21_H_18_O_12_	463.0865	295.0	180278.44
Isoscutellarein 8-glucuronide	C_21_H_18_O_12_	463.0862	313.2	18427.22
Isoscutellarein 8-glucuronide	C_21_H_18_O_12_	463.0870	277.4	1091277.38
Lunularic Acid	C_15_H_14_O_4_	241.0859	442.5	807506.38
Lunularin	C_16_H_18_O_2_	356.2576	447.1	39223.60
Lunularin	C_16_H_18_O_2_	434.1947	454.5	844.54
Lunularin	C_16_H_18_O_2_	241.0859	442.0	690.39
Luteolin 7,3-diglucuronide	C_27_H_26_O_18_	639.1183	232.1	98583.70
Luteolin 7,3-diglucuronide	C_27_H_26_O_18_	639.1185	253.6	50941.54
Luteolin 7,3-diglucuronide	C_27_H_26_O_18_	639.1181	263.0	424450.06
Luteolin 7,3-diglucuronide	C_27_H_26_O_18_	624.3007	226.1	7404.98
Marchantin A	C_28_H_24_O_5_	441.1694	603.5	253393.97
Marchantin A	C_28_H_24_O_5_	441.1700	592.9	2877328.75
Marchantin B	C_28_H_24_O_6_	457.1643	533.1	687680.19
Marchantin C	C_28_H_24_O_4_	425.1747	663.1	281065.75
Marchantin G	C_28_H_22_O_6_	455.1486	548.4	47325.94
Marchantin G	C_28_H_22_O_6_	356.2577	446.7	13174.81
Marchantin G	C_28_H_22_O_6_	340.2637	483.8	15443.49
Marchantin G	C_28_H_22_O_6_	312.1601	516.7	82309.63
Marchantin G	C_28_H_22_O_6_	457.1643	533.5	26383.32
Marchantin K	C_29_H_26_O_7_	487.1737	553.7	29407.29
Paleatin A	C_29_H_28_O_6_	473.1951	562.8	69794.13
Perrottetin E	C_28_H_26_O_4_	427.1900	631.1	145353.53
Pheophorbide A	C_35_H_36_N_4_O_5_	593.2746	944.1	338536.13
Pheophorbide A	C_35_H_36_N_4_O_5_	593.2753	919.3	2613247.25
Thujopsenone	C_15_H_22_O	219.1743	690.7	536620.25
Thujopsenone	C_15_H_22_O	219.1747	661.0	641821.44
Thujopsenone	C_15_H_22_O	219.1743	706.7	44027.77
Thujopsenone	C_15_H_22_O	219.1745	637.5	806036.38
Thujopsenone	C_15_H_22_O	241.1559	644.1	166482.11
Thujopsenone	C_15_H_22_O	241.1559	655.3	27266.09
Thujopsenone	C_15_H_22_O	203.1794	672.8	166351.58
Thunberginol A	C_15_H_10_O_5_	271.0598	409.0	469594.28
Columns are given for the compound name, mass-to-charge ratio (m/z), retention time (RT) in seconds and area under curve (integrated intensity values).				
